# Draft Genome Sequence of *Algoriphagus* sp. Strain NH1, a Multidrug-Resistant Bacterium Isolated from Coastal Sediments of the Northern Yellow Sea in China

**DOI:** 10.1128/genomeA.01555-15

**Published:** 2016-01-14

**Authors:** Dashuai Mu, Jinxin Zhao, Zongjie Wang, Guanjun Chen, Zongjun Du

**Affiliations:** a State Key Laboratory of Microbial Technology, Shandong University, Jinan, People’s Republic of China; b College of Marine Science, Shandong University (Weihai), Weihai, People’s Republic of China

## Abstract

*Algoriphagus* sp. NH1 is a multidrug-resistant bacterium isolated from coastal sediments of the northern Yellow Sea in China. Here, we report the draft genome sequence of NH1, with a size of 6,131,579 bp, average G+C content of 42.68%, and 5,746 predicted protein-coding sequences.

## GENOME ANNOUNCEMENT

In this study, *Algoriphagus* sp. strain NH1 was isolated from coastal sediments of Weihai, which is located on the Yellow Sea (N37°31′04″, E122°01′18″). Analysis of the 16S rRNA gene sequence along with physiological and biochemical characteristics enabled us to identify strain NH1 as an *Algoriphagus* species. The strain has been deposited in the Shandong Infrastructure of Marine Microbial Resources (strain number SDUM203008).

Susceptibility to antibiotics was detected by the disk-diffusion method described by Shah et al. (1). Interestingly, antimicrobial susceptibility testing showed that this strain is susceptible to clindamycin and ofloxacin but is resistant to penicillin, ampicillin, ceftriaxone, cefotaxime, streptomycin, kanamycin, tobramycin, neomycin, gentamicin, tetracycline, biapenem, and lincomycin. Here, we report the draft genome sequence of *Algoriphagus* sp. NH1.

The draft genome of strain NH1 was sequenced by Shanghai Personal Biotechnology Co., Ltd. (Shanghai, China) using Solexa paired-end sequencing technology (2). A library with a fragment length of 400 bp was constructed, and a total of 758,434,670 clean paired-end reads were generated to reach a 132-fold depth of coverage with an Illumina MiSeq (Illumina, USA). The final genome, assembled by SOAPdenovo version 2.04 (3), contained 43 scaffolds consisting of 43 contigs (*N*_50_ = 346,892 bp) with a total size of 6,131,579 bp, and had a mean G+C content of 42.68%.

Gene prediction and annotation were performed with RNAmmer version 1.2 (4), tRNAscan-SE version 1.21 (5), and the Rapid Annotation using Subsystem Technology (RAST) pipeline (http://rast.nmpdr.org) (6). Based on the RAST results, the draft genome contains 5,746 protein-coding genes, giving a coding intensity of 87.23%, the majority of which (3,437; 59.8%) were assigned a putative function, while the remainder were annotated as hypothetical proteins. A total of 1,666 proteins could be assigned to clusters of orthologous groups (COG) families.

Analysis of open reading frames indicated that NH1 possesses at least 50 putative androgen-responsive genes, and 6 putative MarR family transcriptional regulators, which are widely conserved multiple antibiotic resistance regulators that respond to diverse antibiotics, toxic chemicals, and many other important biological processes, were found in the genome (7, 8). Investigation of these genes implies that strain NH 1 has the potential for multidrug resistance in the sediments.

### Nucleotide sequence accession numbers.

This whole-genome shotgun project has been deposited at DDBJ/EMBL/GenBank under the accession number LMXN00000000. The version described in this paper is the first version, LMXN01000000.
